# Elevated IL-18 predicts poor prognosis in critically ill COVID-19 patients at a Brazilian hospital in 2020–21

**DOI:** 10.2217/fmb-2022-0057

**Published:** 2022-09-16

**Authors:** Lucyana LC Coutinho, Caroline N Oliveira, Polianna LMM Albuquerque, Sandra MB Mota, Gdayllon C Meneses, Alice MC Martins, Geraldo BS Junior, Marco AF Clementino, Rafhaella NDG Gondim, Alexandre Havt, Luciano PG Cavalcanti, Juliana NU Yaochite

**Affiliations:** ^1^School of Medicine, Federal University of Ceará, Fortaleza, Ceará, 60430-160, Brazil; ^2^Dr. José Frota Institute, Fortaleza, Ceará, 60025-061, Brazil; ^3^University of Fortaleza, Fortaleza, Ceará, 60811-905, Brazil; ^4^Faculty of Pharmacy, Dentistry & Nursing, Federal University of Ceará, Fortaleza, Ceará, 60430-370, Brazil

**Keywords:** COVID-19, IL-18, immune response, inflammation, innate immunity

## Abstract

**Background:** A dysregulated inflammatory response contributes to decline in patients with COVID-19. This cross-sectional study evaluated biomarkers of unvaccinated patients admitted to the intensive care unit of a hospital in Fortaleza, Brazil. **Methods:** Twenty cytokines were quantified upon hospital admission; clinical and laboratory data were analyzed, as well as sociodemographic data, to search for an association with clinical outcomes, including fatal (n = 40) or recovered cases (n = 38). **Results:** Fatal cases exhibited significantly higher levels of IL-18 (p = 0.009); deceased patients were older (p = 0.0001), had a lower number of platelets (p = 0.0063) and higher neutrophil–lymphocyte ratio (p = 0.0230) than those who recovered. **Conclusion:** These findings indicate that IL-18 is a possible marker to predict poor prognosis in critically ill patients with COVID-19.

As of 28 February 2022, more than 444 million confirmed cases of COVID-19, caused by the SARS-COV-2 virus, and more than 6 million deaths had occurred, according to data from the World Health Organization COVID-19 Dashboard. It is now the most severe pandemic of the 21st century. Since the first cases were reported in December 2019, an attempt has been made to understand how the disease develops and which factors are associated with the broad spectrum of responses and outcomes of those infected [1].

The immune system plays a central role in determining the course of viral infections. Unregulated inflammatory response, whether insufficient or exacerbated, triggers a lower capacity to resolve the disease, leading to harmful effects on the body. This unregulated inflammation is called cytokine storm and is characterized by exacerbated production of cytokines from activated macrophages, dendritic cells, neutrophils, monocytes, NK cells, T and B lymphocytes and endothelial and epithelial cells [2]. IL-18 belongs to the IL-1 family and is the most crucial proinflammatory cytokine produced by macrophages at the beginning of the inflammatory response. It is responsible for the regulation of both T-helper response 1 (Th1) and 2 (Th2), in addition to being associated with the exacerbation of inflammation and tissue damage [3,4].

Investigating biomarkers that guide the prognosis of the SARS-CoV-2 is crucial because such knowledge would help in early and aggressive patient therapy [5]. This cross-sectional study aimed to evaluate serum markers in unvaccinated patients admitted to the intensive care unit (ICU) at a reference hospital for COVID-19 in Fortaleza, northeastern Brazil, in 2020–21.

## Methods

### Study setting & patient selection

This cross-sectional study was conducted at the ICU of Dr Jose Frota Institute (IJF), a reference hospital for COVID-19, in Fortaleza, Brazil, from June to August 2020 and January to February 2021. The reported hospital was the primary reference in assisting COVID-19 patients requiring ICU. All patients (n = 78) were from the state of Ceara, and most from the capital city of Fortaleza (n = 65). All patients included in this study were admitted to the ICU, tested positive for SARS-CoV-2 infection and had not been vaccinated. The main factor considered to admit the patient to the ICU was the severity according to the Simplified Acute Physiology Score 3 [6] and the need for invasive support such as vasoactive drugs and/or mechanical ventilation. Almost all patients were admitted on the 8–10th day after symptom onset. Samples were collected upon admission after obtaining consent from the patients or their families.

Diagnosis was performed by a rapid test based on antigen detection or by detection of viral RNA from nasopharyngeal swabs using reverse transcription polymerase chain reaction (RT-PCR). The serum samples were collected for each patient on the first day of hospital admission in tubes without anticoagulants. Samples were centrifuged, aliquoted and stored in an ultra freezer at -80 °C until analysis. Quantification of serum inflammatory biomarkers GM-CSF, MCP-1, TNF-α, IFN-α and IFN-γ, and IL-1β, IL-10, IL-12p70, IL-13, IL-17A, IL-18, IL-2, IL-21, IL-22, IL-23, IL-27, IL-4, IL-5, IL-6 and IL-9 was performed using a custom kit for 20 cytokines named Human ProcartaPlex Multiplex Immunoassay (Thermo Fisher Scientific, MA, USA). The measurement was performed using the MAGPIX System (Merck, MA, USA) based on Luminex xMAP technology, according to the manufacturer's instructions.

### Inclusion criteria

The eligibility criteria of the study were age ≥18 years; admission to ICU; confirmatory laboratory tests for the presence of SARS-COV-2 (RT-PCR and/or rapid antigen test) and manifestation of severe acute respiratory syndrome.

### Exclusion criteria

Exclusion criteria adopted by this study were individuals with inconclusive tests for COVID-19, pregnant women, transplanted patients, individuals using immunosuppressive drugs and/or patients undergoing chemotherapy treatment.

### Sample size

Serum samples were collected on the first day of admission from 78 individuals admitted to the ICU with a confirmed diagnosis of SARS-COV-2 infection. In the first study interval (June–August 2020), 65 patients were randomly selected. From the second study interval (January–February 2021), 15 patients were randomly selected. However, two samples had to be excluded from the analysis because they did not meet the inclusion criteria. After selection, the patients chosen were divided according to the clinical outcome of discharge and death. Thus, the total number of participants in this study comprised 40 fatal cases and 38 recovered cases.

### Data collection

Sociodemographic and laboratory data were collected from electronic medical records available in the Ars Vitae system of the IJF. Only the laboratory results from the day of admission were included in the study.

### Study objective

The study aimed to evaluate serum biomarkers of patients with SARS-CoV-2 admitted to the ICU relating to clinical outcomes of recovered or fatal.

### Statistical measure

GraphPad Prism software version 8.0.1 was used for statistical analysis and data presentation. The Shapiro–Wilk test was applied to evaluate the normal or nonnormal distribution. Nonparametric categorical variables were expressed as absolute and relative frequency and tested for statistically significant differences using the Pearson chi-square test and Fisher test. Nonparametric continuous variables were presented as the median and interquartile range (IQR); data were analyzed by the Mann–Whitney *U* test. To explore the correlations between IL-18 and GM-CSF, IL-22, IL-23, age, platelets and lymphocytes, Spearman correlation analysis was performed due to the non-normal distributions of these variables. The results were considered statistically significant whenever the p-value was lower than 0.05 (p < 0.05).

### Ethical approval

Informed consent was obtained from each involved participant. This study was approved by the National Research Ethics Commission (CONEP, protocol no. 4.026.888) and by the Research Ethics Committee of the Federal University of Ceará (PROPESQ/UFC, protocol no. 4.346.280).

## Results

Among the 78 patients in the study, 40 (51.2%) died and 38 (48.8%) recovered. Most individuals were men (64.1%), with a median age of 59 (range: 22–97) years. Systemic arterial hypertension was the most frequent comorbidity (30.7%). Patients with a history of the cerebrovascular accident showed statistical significance compared with patients without comorbidity. Sociodemographic and clinical details are shown in Table 1.

**Table 1.** Sociodemographic, clinical and laboratory characteristics of patients with severe COVID-19 in the intensive care unit of a tertiary hospital in the state of Ceara, northeastern Brazil.

Characteristics	Total (n = 78)	Fatal cases (n = 40)	Recovered (n = 38)	p-value
**Men** ^†^	50 (64.1%)	28 (70%)	22 (57.9%)	0.3961
Women^†^	28 (35.9%)	12 (30%)	16 (42.1%)	0.4497
Age (years)^‡^	59 (22- 97)	69.50 (22–97)	41 (22–82)	**0.0001**
Comorbidities^‡^				
Systemic arterial hypertension	24 (30.7%)	14 (35%)	10 (26.3%)	0.4142
Obesity/overweight	12 (15.4%)	5 (12.5%)	7 (18.4%)	0.5637
Diabetes mellitus 1 and 2	15 (19.2%)	11 (27.5%)	4 (10.5%)	0.0707
Heart diseases	13 (16.7%)	7 (17.5%)	6 (15.8 %)	0.7815
Lung diseases	9 (11.5%)	4 (10%)	5 (13.2%)	0.7389
Cerebrovascular accident	6 (7.7%)	6 (15%)	0	0.0143
Former smokers	4 (5.1%)	3 (7.5%)	1 (2.6%)	0.3173
Former alcoholics	3 (3.8%)	3 (7.5%)	0	0.0833
Other	9 (11.5%)	5 (12.5 %)	4 (10.5%)	0.7389
No comorbid adherence	32 (41%)	13 (32.5%)	19 (50%)	0.2888
Days of hospitalization (n)^‡^	12 (1–83)	10 (1–67)	14.50 (3–83)	0.2422
C-reactive protein^‡^	133.1 (0.7–515)	142.3 (0.7–488.8)	109.7 (2.1–515)	0.3716
D-dimer^§^	2.420 (0.23–33.01)	2.440 (0.56–25.02)	2.370 (0.23–33.01)	0.3257
Leukocytes^‡^	13,615 (3730–35,660)	14,140 (3,820–29,560)	13,050 (3730–35,660)	0.5277
Neutrophils^‡^	12,235 (3148–31,808)	12,551 (3202–27,490)	10,718 (3148–31,808)	0.3267
Lymphocytes^‡^	998 (231–4478)	805 (306–2.874)	1.079 (231–4478)	0.0714
Neutrophil-lymphocyte ratio	11.79 (11.65–48)	12.79 (14.44–48)	10.33 (11.65–40.17)	**0.0230**
Monocytes^‡^	533 (0–2.088)	452.5 (76–2.088)	554.5 (0–1.676)	0.1694
Platelets^‡^	235,500 (48,000–564,000)	193.500 (48,000–511,000)	274,500 (68,000–564,000)	**0.0063**

† Absolute frequency (relative).

‡ Median (interquartile range).

§ Fatal cases, n = 32; recovered cases, n = 33.

p < 0.05.

When associating the categorical variables of comorbidities, the Pearson and Fisher chi-square tests showed a statistically significant association only between the groups with the cerebrovascular accident (CVA) and without comorbidities (p = 0.0076) for both recovered and fatal cases.

Fatal cases were older (median: 69.50; IQR: 22–97), had lower platelet concentration (median: 193.500; IQR: 167.000–256.000), higher neutrophil–lymphocyte ratio (NLR; median: 12.79; IQR: 14.44–48) and higher concentration of IL-18 (median: 139.2; IQR: 36.64–3.361.0) compared with recovered cases. Spearman correlation analysis showed that age (r = 0.003, p > 0.05), gender (r = -0.1300, p > 0.05), platelet count (r = 0.04, p > 0.05) and lymphocyte count (r = -0.22, p > 0.05) had no statistical significance with IL-18 levels.

**Figure 1.**
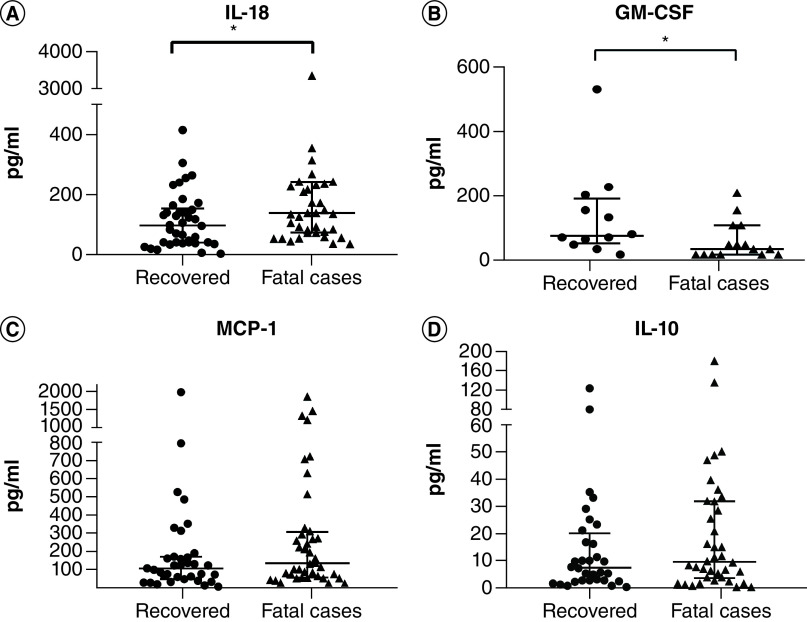
Comparison of IL-18, MCP-1, IL-10 and GM-CSF cytokine values between fatal and recovered cases, respectively. Mann–Whitney test (*p < 0.05). **(A)** IL-18 (p = 0.009; n = 40, 38). **(B)** GM-CSF (p = 0,0187; n = 16, 12). (**C)** MCP-1 (p = 0.1863; n = 40, 35). **(D)** IL-10 (p = 0.3584; n = 38, 32).

Some cytokines showed results below the lower limit of quantification. Therefore, only samples with detectable levels were used in the analyses. Despite the small sample size, GM-CSF (n = 28), IL-22 (n = 19) and IL-23 (n = 8) values were significantly lower in fatal cases (p = 0.0187, 0.0473 and 0.0313, respectively). Increase in IL-18 was not correlated (p > 0.05) with changes in GM-CSF (p = 0.2826), IL-22 (p = 0.3268) or IL-23 (p = 0.2417). Despite nonsignificant results, IL-10 and MCP-1 are graphically presented as they were detected in most samples. Supplementary Table 1 demonstrates the medians and amounts of samples with detected levels for each cytokine. Figure 1 shows the comparisons of the cytokines IL-18, IL-10, MCP-1 and GM-CSF levels from fatal cases and recovered groups.

## Discussion

### IL-18 is elevated in fatal cases of COVID-19

In the present study, IL-18 levels were higher in patients who died compared with discharged patients with severe COVID-19. Recent studies show that the proportion of proinflammatory and anti-inflammatory cytokines can be used for risk stratification in hospitalized patients with COVID-19. IL-18 has been reported as a predictor of infection severity [1,7–9]. Considering IL-18 biological functions, it was first reported to induce interferon-γ activity [10]. IL-18 regulates the Th1 and Th2 responses, and its increased production can lead to an exacerbated inflammatory state [3]. In COVID-19 patients, IL-18 has been studied for its role as a component of inflammasomes [11]. Inflammasomes are cytosolic protein complexes important in the innate immune response against pathogen-associated molecular patterns. From the activation of inflammasomes, proinflammatory cytokines such as IL-1β and IL-18 are activated and released into the extracellular environment, leading to pyroptosis (cell death by lysis) and inflammation. When this process is overactivated, exacerbated inflammation triggers the disease aggravation. Studies show that NLRP3 (NOD-, LRR- and pyrin domain-containing protein 3) inflammasome is highly expressed in patients with COVID-19, being a potential marker of disease severity [11,12].

Furthermore, IL-18 is also associated with worsening and mortality in other viral diseases such as influenza and dengue [7]. Together with other cytokines, increased expression of IL-18 contributes to macrophage activation syndrome, a condition seen in autoimmune diseases and influenza. Previous studies revealed similarities in the pathogenesis of COVID-19 and macrophage activation syndrome [3,12].

### Other relevant cytokines: GM-CSF, IL-10 & MCP-1

GM-CSF is a proinflammatory cytokine produced by macrophages, T cells, fibroblasts, endothelial cells and epithelial and tumor cells, with most of the production occurring in inflammatory sites. GM-CSF levels are low or undetectable under normal conditions. However, any immune trigger can rapidly increase its concentration [13]. Elevated levels of GM-CSF were found in the bronchoalveolar fluid of patients with SARS compared with healthy controls [14]. In addition, the use of GM-CSF and this cytokine inhibitor (anti-GM-CSF monoclonal antibodies) as a therapeutic strategy in COVID-19 has been reported. However, a dual role is related to GM-CSF, depending on the stage of the disease and the amount of this molecule [15].

In this study, the concentration of GM-CSF was lower in fatal cases compared with recovered patients, suggesting a positive role of a nondelayed or exacerbated response of this cytokine in the recovery of patients, associated, for example, with differentiation and activation of alveolar macrophages that contribute to viral clearance [16].

IL-10 is an anti-inflammatory cytokine described to be elevated in patients with severe COVID-19 [1,9]. Because of the inflammatory context of these individuals, it is believed that the increase in IL-10 would be a compensatory response of the body in an attempt to quell the inflammatory process. Elevated IL-10 was related to the T-cell exhaustion from the overactivation and proliferation of these cells [16]. MCP-1 is responsible for the recruitment of monocytes and macrophages, contributing to exacerbated inflammation and thromboembolic disorders when occurring in high amounts in the body. In COVID-19, its increase in critically ill patients has also been reported [1,17].

### Comorbidities, age, platelets & NLR: significant in fatal cases

Several infectious agents are considered to be risk factors for CVA development. A systematic review by Kazemi *et al*. [18] showed that SARS-CoV-2 infection could be correlated with CVA. The putative mechanisms that may explain this relationship between stroke and COVID-19 include coagulopathies (especially thrombophilia), the expression of angiotensin-converting enzyme in CNS cells and interaction with SARS-CoV-2, endothelial dysfunction, microthrombosis and activation of the proinflammatory cytokine cascade. Klok *et al*. [19] reported that thrombotic complications occurred in 31% of critically ill ICU patients with SARS-CoV-2. Our study corroborates these findings, as it shows that patients with severe COVID-19 and stroke had a higher risk of death (p = 0.0143) than those without comorbidities.

Another significant finding of our study, consistent with other studies, is that older patients showed higher death rates (p = 0.001). Tjan *et al*. [8] reported that asymptomatic or mildly symptomatic patients were significantly younger than those with severe disease. Age-associated severe infection seems to be due to immunosenescence, followed by comorbidities and age-related variation in the expression of angiotensin converting enzyme 2 [20].

Patients with COVID-19 pneumonia exhibit coagulation abnormalities, often with mild thrombocytopenia. On average, patients with severe diseases have a lower platelet count than those with nonsevere disease [21]. Thrombocytopenia on admission in patients with COVID-19 was associated with 4.24-fold increased mortality risk [1]. Similarly, our results showed lower platelet count in individuals who died than in the discharge group. In agreement with Liu *et al.* [22], which showed that NLR, an inflammatory marker, is an independent risk factor for the individuals with COVID-19 in hospital mortality, the NLR of patients in our study was significantly higher in fatal cases. Thus, inflammatory and regulatory responses play a significant role in controlling the infection caused by SARS-CoV-2 and can also predispose to severe pulmonary and systemic manifestations in certain individuals. Factors associated with age, comorbidities and alteration in blood components may contribute to the poor prognosis in critically ill COVID-19 patients.

## Conclusion

This study evidenced the prediction of the outcomes of COVID-19 patients from ICU through pro-inflammatory cytokine evaluation. Our results demonstrate that the concentration of IL-18 was higher in individuals with the clinical outcome of death. In addition, sociodemographic and laboratory characteristics such as advanced age, history of CVA, low platelet count and high NLR were related to fatal cases. Among the limitations of our study are the small sample size, absence of a control group without COVID-19 and with COVID-19 but not admitted to the ICU, and nonanalysis of the follow-up of patients with a serial sample.

Recognizing differences between high- and low-risk individuals with poor prognosis through biomarkers can help determine the best preventive and treatment therapy, in addition to helping to understand the pathogenesis of the disease.

**Summary points**

- Immune characteristics of individuals infected with COVID-19 have been extensively investigated.
- Unregulated inflammatory response, whether insufficient or exacerbated, triggers a lower capacity to resolve the disease, leading to harmful effects on the body.
- It is essential to investigate biomarkers that guide the prognosis of the SARS-CoV-2 infection; such knowledge helps in early and aggressive intervention in patient therapy.
- This cross-sectional study aimed to evaluate serum markers as predictors of poor clinical outcomes in unvaccinated patients admitted to the intensive care unit at a reference hospital for COVID-19 in Fortaleza in northeastern Brazil. The secondary aims were to evaluate the sociodemographic, clinical and laboratory characteristics of these patients.
- Seventy-eight patients met the inclusion criteria and were divided into two groups: fatal cases (n = 40) and recovered (n = 38).
- Sex, age, comorbidities, hospitalization length, C-reactive protein values, D-dimer, leukocytes, neutrophils, lymphocytes, neutrophil–lymphocyte ratio, monocytes and platelets, and levels of 20 cytokines were compared between the groups.
- Fatal cases were older and had lower platelet concentration, higher neutrophil–lymphocyte ratio and higher IL-18 concentration than recovered patients. Correlation analysis showed that age, sex, platelets, lymphocytes, GM-CSF, IL-22 and IL-23 had no statistical significance with IL-18.
- This study shows that elevated IL-18 predicts poor prognosis in critically ill COVID-19 patients. Combined with other studies, these findings can help determine the best preventive and treatment therapy.
