# Supplementary material for: Elevated IL-18 predicts poor prognosis in critically ill COVID-19 patients at a Brazilian hospital in 2020–21
Source: Future Microbiol. 2022 Sep 16:10.2217/fmb-2022-0057. doi: 10.2217/fmb-2022-0057 (PMC9488117; doi:10.2217/fmb-2022-0057)
Supplement: Supplementary file 1 [file Supplementary-material-review.docx]

**Supplementary material**

| **Cytokine** | **Fatal cases** | | **Recovered** | | **P value** |
| --- | --- | --- | --- | --- | --- |
|  | **pg/ml** | **n**** | **pg/ml** | **n**** |  |
| **GM-CSF** | 35.05 (18.39-209.4) | 16 | 76.27 (18.39-531.6) | 12 | **0.0187*** |
| **IL-2** | 53.50 (9,804-87.23) | 4 | 38.08 (20.74-90.98) | 11 | 0.9700 |
| **IFN-a** | 0.8300 (0.173-3,272) | 9 | 0.5820 (0.006-6.269) | 9 | 0.4227 |
| **IL-4** | 6.067 (0.7330-68.54) | 17 | 12.53 (0.7330-203.5) | 14 | 0.1223 |
| **IFN-g** | 16.25 (0.7190-215.9) | 37 | 14.80 (1,928-158.7) | 26 | 0,6592 |
| **IL-5** | 10.54 (1,918-78.13) | 20 | 21.45 (4,876-310.7) | 18 | 0.1652 |
| **IL-6** | 165.3 (28.26-4,014.0) | 21 | 202 (2,056-1,200.0) | 16 | 0.6328 |
| **IL-9** | 41.64 (2.518-118.2) | 21 | 20.07 (2.518-103.3) | 15 | 0.4035 |
| **IL-10** | 9,630 (0.344-179.9) | 38 | 7,458 (0.344-123.6) | 32 | 0.3584 |
| **IL-12P70** | 1,062 (0.09-8,425) | 33 | 0.8050 (0.09-30.91) | 24 | 0.8690 |
| **IL-17A** | 3,395 (1,158-32) | 30 | 7,329 (1,158-73.84) | 20 | 0.0917 |
| **IL-13** | 7,456 (3.157-42.12) | 20 | 11.78 (3.157-101.2) | 14 | 0.2606 |
| **IL-18** | 139.2 (36.64-3,361.0) | 40 | 98.28 (4.606-414.4) | 38 | **0.009*** |
| **IL-21** | 16.03 (3.220-547.1) | 21 | 11.08 (0.2440-220.1) | 15 | 0.8306 |
| **IL-22** | 81.28 (8.209-966.2) | 12 | 168.8 (69.96-475.2) | 7 | **0.0473*** |
| **IL-23** | 54.11 (23.18- 85.04) | 2 | 44.13 (14.16-271.1) | 6 | **0.0313*** |
| **IL-1b** | 12.28 (6,310-53.88) | 10 | 12.41 (6,310-171.4) | 12 | 0.3157 |
| **IL-27** | 26.10 (2047-197.9) | 16 | 51.93 (2047-487.4) | 14 | 0.1012 |
| **MCP-1** | 134.7 (23.33-1.854.0) | 40 | 106.6 (5.886-1.973.0) | 35 | 0.1863 |
| **TNF-a** | 17.80 (1.389-118.9) | 25 | 20.96 (1.389-185.4) | 20 | 0.8962 |

**Supplementary Table 1. Values of the 20 cytokines ​​from fatal cases and recovered. Median (interquartile range).**

* p < 0.05
** n= number of samples with detectable amounts of cytokine

Legend: granulocyte-macrophage colony-stimulating factor human (GM-CSF), monocyte chemoattractant protein-1 (MCP-1), tumor necrosis factor (TNF-α), interferons (IFN-α and IFN-γ) and interleukins (IL-1β, IL-10, IL-12p70, IL-13, IL-17A, IL-18, IL-2 , IL-21, IL-22, IL-23, IL-27, IL-4, IL-5, IL-6, IL-9).
